# Including the ‘Spiritual’ Within Mental Health Care in the UK, from the Experiences of People with Mental Health Problems

**DOI:** 10.1007/s10943-017-0502-1

**Published:** 2017-10-24

**Authors:** R. Forrester-Jones, L. Dietzfelbinger, D. Stedman, P. Richmond

**Affiliations:** Tizard Centre, School of Social Policy, Sociology and Social Research, University of Kent, Canterbury, UK

**Keywords:** Spirituality, Mental health, Social support, Qualitative

## Abstract

Spirituality as a dimension of quality of life and well-being has recently begun to be more valued within person-centred treatment approaches to mental health in the UK. The aim of this paper is to provide indicators of the extent to which accessing a spiritual support group may be useful within mental health recovery from the view point of those in receipt of it. The study design was a small-scale exploratory study utilising mixed methods. Quantitative methods were used to map the mental health, general well-being and social networks of the group. These were complimented by a semi-structured open-ended interview which allowed for Interpretative Phenomenological Analysis (IPA) of the life-history accounts of nine individuals with mental health problems who attended a ‘spirituality support group’. Data from unstructured open-ended interviews with five faith chaplains and a mental health day centre manager were also analysed using thematic analysis. The views of 15 participants are therefore recounted. Participants reported that the group offered them: an alternative to more formal religious organisations, and an opportunity to settle spiritual confusions/fears. The ‘group’ was also reported to generally help individual’s subjective feelings of mental wellness through social support. Whilst the merits of spiritual care are appealing, convincing services to include it within treatment may still be difficult.

## Introduction

Generally within society, ‘spirituality’ and ‘religion’ tend to be used interchangeably. Social scientists, however, usually separate the two, using the term spirituality to describe faith in ‘something’ [e.g. a supreme power (see King et al. 1995; King and Koenig 2009; Baker 2003)] and religion as a more organised social phenomena (Thoresen 1998) with ‘being religious’ understood as a state of observance or obedience to beliefs, practices and rituals directed towards a specific being or power (Zinnbauer et al. 1997). Caring for the spiritual aspect of a person’s life has been considered an important part of nursing the sick since ancient times (Narayanasamy 1999). According to Miller and Thoresen (2003), however, research on spirituality and mental health has been hindered by a conceived wisdom generally held by proponents of the medical model, that spirituality is ‘beyond scientific measurement’ (Powell 2001).

Nevertheless, notwithstanding research reporting that spiritual beliefs can lead to detrimental effects linked to mental health problems including anger towards ‘supreme beings’ or religious organisations (Seybold and Hill 2001; Cohen and Koenig 2004), a growing body of international literature demonstrates a generally positive relationship between spirituality and mental health (see Hill et al. 2000, Hill and Pargament 2003; Macmin and Foskett 2004, Baetz et al. 2006, Coyte et al. 2007; Koenig 2005; Bonelli and Koenig 2013). Such studies have explored how spirituality or spiritual involvement affect depression (Westgate 1996), anxiety (Graham et al. 2001), decision making, and resilience to traumatic events (Pargament et al. 1988; Koenig 2010), as well as whether, as a life domain, spirituality helps to buffer life-stress (Hayman et al. 2007). Lindgren and Coursey (1995) in a survey of 28 patients with serious chronic mental health problems found that more than half the sample found spirituality to have been significant to their recovery.

### Spirituality as a Protective Factor in Suicide

A range of studies have also looked at spirituality or affiliation to spiritual or religious groups in relation to suicide (Dervic et al. 2004) and suicide attempts (Rasic et al. 2009, 2011). Corroborating Durkheim’s (1897) seminal work on suicide and religious affiliation, Nisbet et al. (2000) reported that the suicide rate is four times lower in people who attend religious activities. In a study of 1610 older adults with depression, Chen et al. (2007) also found that those attending religious activities on a fairly regular basis, who reported having a spiritual faith, were significantly less likely to have suicidal ideation and emotional distress compared to non-attendees. Finding higher rates of suicide among those who have no religious affiliation, studies have also posited that association with a religion may act as a protective factor against suicide (Pescosolido and Georgianna 1989, Lester 2000; Lester 2012)). All of these studies, however, leave a question mark as to what exactly it is about religious association which is protective: the shared community of social relationships, or the religious social mores or ‘rules’ (e.g. the Ten Commandments) which prevent behaviours (e.g. drug abuse) at risk of leading to psychiatric disorder and suicide (e.g. drug abuse)?

### Spirituality as an Agent of Recovery

Spirituality has been found to be an important component in the recovery of severe mental illness (Corrigan et al. 2003). During the past two decades, research has connected spirituality (including a sense of meaning and participation in faith communities) to a variety of benefits, including increased hope, well-being, self-esteem, social supports, motivation towards growth, as well as decreased depression, anxiety, and substance abuse (see Snider and McPhedran 2014). As a result, practitioners and academics are increasingly recognising spirituality as a relevant dimension of recovery (Fallott 2001; Whitley and Drake 2010, Starnino and Canda 2014; Carlisle 2016).

### Policy and Practice: The Spiritual Gap

Despite the evidence presented above regarding the merits of spirituality in relation to mental health, a systematic policy review using UK government databases identified policy and government directives as regards the spiritual care of people in general, and more specifically of people with intellectual disabilities or mental health problems (Sango and Forrester-Jones 2014). Further, although all UK NHS trusts are required to provide spiritual support for patients staff and relatives through chaplains/faith representatives [NHS 2001; 2003 (updated 2015)], people with mental health problems have tended to be treated primarily with medication. In line with the medical model, psychiatrists are likely to pathologise the patient’s spiritual experiences (Awara and Fasey 2008), absenting spiritual matters from treatment models (Genic 1994); Foskett (1996) calling religion ‘psychiatry’s last taboo’ (cited in Macmin and Foskett 2004). This is despite evidence that cognitive behavioural psychotherapies which include patient’s spiritual convictions can help treat depression and anxiety (Razali et al. 2002; Faulkner and Layzell 2000).

Only recently has spirituality started to be viewed as a vital dimension of holistic practice and person-centred care (Barrera et al. 2012) with clinicians being trained in Mindfulness, (a form of meditation based on the Buddhist tradition), and more recently Mindfulness Stress Reduction, and Mindfulness Cognitive Therapy (see Williams and Penman 2011), and to incorporate spiritual matters within therapeutic interventions (Bögels et al. 2010; Paulik et al. 2010). Spirituality is also now acknowledged as a support mechanism for mental health professionals (see de Zoysa et al. 2014). Cohen and Koenig (2004) and Koenig (2013), however, caution clinicians against prescribing religious practices or imposing their own religious beliefs on patients.

Nevertheless, despite this progress, a ‘spiritual gap’ (Thoresen 1999) between mental health patients and health professionals appears to remain. D’Souza’s (2002) study reported that although the majority of mental health patients found spirituality important, and wanted their doctors/therapists to take their spiritual needs into account during treatment, this did not routinely happen. Possible reasons for such ‘spiritual oversight’ have been posited as clinicians being generally less spiritual then their patients (Hill et al. 2000), and the absence of precise measures of spirituality/religiosity (Miller and Thoresen 2003). This means that any attempt at ‘curing’ spiritual issues within the medical model is bound to be beset with problems. It is also unclear as to whether it is the spiritual element of communal spiritual activity which helps avoid or aid recovery of mental ill health, or the social environment provided by a spiritual community (Hadzic 2011:223). In Macmin and Foskett’s (2004) grounded theory study, service users/survivors participated as both interviewers and interviewees. Analysis of 11 interviews provided evidence of the significance of spirituality for some participants, as well as how difficult it was for this aspect of their lives to be taken seriously by mental health professionals. In Awara and Fasey’s (2008) study of 43 patients attending a psychiatric outpatient service for 3 months, over half (54%) felt that their spiritual or religious beliefs improved their coping strategies (p. 183). Despite this, half the sample interviewed reported that they felt that their beliefs were not regarded as important by the service. This may be due to the fact that psychiatrists and psychologists still receive little or no training in how to deal with spiritual issues which may arise during clinical practice (Sansone et al. 1990) despite research advocating inter-professional training (Carpenter et al. 2006). Bonelli et al. (2012) state that further studies on this topic are required.

## Aim of Study

The aim of this small-scale exploratory study was to discover whether and in what ways a spirituality support group (SSG) mediated mental well-being from the view point of the attendees. To this end, the following research questions were addressed:

1. What motivated people with mental health problems to join the SSG?
2. What were their experiences of it?
3. Did they feel that the group helped in their recovery process?

## Method and Materials

### Design

The study design was explorative, utilising a mixed methods approach. Combining quantitative and qualitative methods enabled a more complete and complementary understanding of the social phenomena under investigation (Morgan 2007).

### Participants

The purposively drawn sample included nine individuals with a mental health problem who were attending a spiritual support group organised by the local mental health service. Inclusion criteria were based on a mental health diagnosis; continuous contact with the local psychiatric service for more than 1 year; and referral to the mental health community day centre as part of their recovery programme. Five mental health chaplains were also purposively drawn for the study. The manager of the day centre where the SSG took place was also interviewed. We therefore had in-depth data for a total of fourteen individuals.

### Location of Study

The Spiritual Support Group was held at a community day centre, situated in an ordinary house. The SSG was run by the five mental health chaplains although one particular chaplain tended to facilitate the group most weeks. The aims of the SSG were specified as helping mental health service users to:

- understand and accept who they were as individuals;
- pursue positive relationships in the community;
- take delight in the arts and the natural environment;
- explore some of the mysteries of human life (Stedman 2006:1).

The ten-week SSG programme was structured as a formative module with hour-long sessions deliberately kept flexible so as to enable the group to develop naturally within a planned framework. Group discussions were confidential except where the facilitator considered disclosures to be relevant to service user care. There was no expectation of ‘homework’ though handouts were provided each week indicating the plan of activities for the following session. Although pastoral care was provided, no attempt was made to address clinical treatments or to develop psycho-therapeutic relationships. Whilst the group was mainly discussion based, music, poetry, stories and pictures were also used to give participants an opportunity to access different sides of their spirituality. One of the chaplains often played his guitar, either singing to the members or leading group singing. Sometimes the group met at local coffee bars and parks, providing an alternative environment from the day centre. Whilst the chaplains all had a background within various denominations of the Christian faith, there was no expectation that the group would be Christian in essence—rather discussions centred on what spirituality meant to individual members.

### Quantitative Measures

The *Krawiecka Rating Scale* (Krawiecka et al. 1977) provided an objective rating of the mental state of the sample, and the *General Well*-*Being Scale* (Goldberg and Williams 1988) was used to detect psychiatric conditions among the group. The *Social Network Guide* (Forrester-Jones and Broadhurst 2007) provided a profile of individuals’ social lives including the structure of their networks [size (or number of people attached to them), and membership (who the contacts actually were)] and the support behaviours provided by network members. Group scores of psychiatric state, general well-being and social network size allowed us to present a broad profile of the group who shared particular characteristics and to help contextualise the qualitative vignettes. Reporting at this level also meant that we could broadly compare the group’s experiences, particularly in relation to social networks, with those reported in other studies, thus adding to the overall discussion of how useful a spiritual support group might be to individuals with mental health problems.

### Qualitative Measure

A *semi*-*structured open*-*ended interview* was devised to allow life histories to be provided and to explore whether and in what ways the SSG subjectively helped them in their recovery process. An unstructured *open*-*ended interview* was also used to explore the extent to which the day centre manager’s and chaplains’ perceptions of the SSG matched those of the mental health sample. All of the interviews lasted around 90 min each, and all but two participants consented to their interviews being audio-recorded. The researcher also engaged in participant observation, attending the SSG each week for 1 year.

### Analysis

Since the aim of the study was to understand how participants made sense of their experiences of the SSG, Interpretative Phenomenological Analysis (IPA) was utilised (Brocki and Wearden 2006; Flick 2006). IPA is an inductive process that seeks to produce themes from the data rather than extracting data based on a preconceived theory (Smith et al. 2009). All of the transcribed interviews were read through several times and independently coded by the first two authors. Codes were compared for reliability and the data re-organised jointly with categories and themes chosen for their idiographic nature, with the aim of capturing the full range of experiences and interpretations of all participants. Fieldwork notes were also analysed as a check on emergent themes. Transcribed interviews from the chaplains and manager were subjected to thematic analysis again by both researchers until saturation of themes common to all of the participants was reached. Quantitative data were analysed using SPSS.

### Ethical Issues and Study Limitations

The research proposal was ethically peer reviewed by a University ethics committee (ref:5/05) and gained a favourable ethical opinion from the UK National Health Service Research Ethics Committee (ref: 05/Q1803/54). Each potential participant was given an information sheet explaining the nature, reason for and voluntary aspect of the study; a consent form to sign; and a complaints form. In line with the NHS Support Unit advice to pay unwaged research participants for their time (Hanley et al. 2000), each individual was paid £10 (the amount determined by the project funding) post-interview so as not to compromise voluntariness. In order to maintain confidentiality, pseudonyms are used in this paper. The research was limited in scope/participant number due to the very limited research funding available (£500).

## Findings

### Participant Characteristics

The participants (*n* = 9; 4 males) had all been in contact with psychiatric services for more than a year and had been referred to a mental health community day centre as part of their recovery programme. The average age was 37 years (see Table 1), and all were White, reflecting the study location (Southern England, UK). All were unemployed and had been attending the SSG, which was organised as part of the optional mental health day centre activities, for on average 6 months. Whilst people of Hindu, Muslim, Sikh or Jewish faith were not represented in the sample, a number of people held New Age or Atheist as well as Christian beliefs. The five chaplains (1 was female) were all white British and had an average age of 52 years. They had all been practicing as mental health chaplains for more than 5 years. The day centre manager had been in post for 6 years.

**Table 1:** Characteristics of participants

Pseudonym	Gender	Age	Ethnic origin	Diagnosis
Peter	M	28	White	S-AD
Susan	F	23	White	Depression/BPD
Helen	F	47	White	Depression/anxiety
Laura	F	57	White	Depression
Kathy	F	27	White	BPD/depression/ED
Simon	M	59	White	Depression
Jane	F	26	White	Depression
David	M	61	White	Depression/anxiety
John	M	41	White	Depression

*S-AD* Schizo-affective disorder; *BPD* borderline personality disorder; *ED* eating disorders

### General well-being of SSG Attendees

The average general well-being score was 2.96 (range 2.21–3.50) indicating a group who were experiencing moderate levels of psychiatric disorder. Table 2 shows a breakdown of the proportion of the sample scoring 4 (maximum well-being) for each domain. Only 4 out of the 9 participants reported not constantly feeling under strain, and only 3 felt their concentration was good, they were not losing sleep over worry, were able to face up to their problems, could enjoy normal day-to-day activities and generally felt happy. Only 1 person said they thought they were capable of making decisions or that they had been feeling happy and not depressed; unsurprising results, given the diagnosis of the individuals.

**Table 2:** General well-being

General well-being domains	Number of people scoring 4 (max well-being on a scale of 1–4) *N* = 9
Good concentration	3
Not losing sleep due to worry	3
Feel I am playing a useful part in things	5
Capable of making decisions	1
Not constantly under strain	4
Feel I can overcome difficulties	2
Been able to enjoy normal day-to-day activities	3
Been able to face up to problems	3
Been feeling happy and not depressed	1
Have confidence in self	5
Do not feel a worthless person	2
Feeling happy all things considered	3

However, over half of all participants reported confidence in themselves and, half also said that they were playing a useful part in things including the SSG, Susan stating *‘each member is as important as everyone else…and everyone gets a say’*.

### Mental State of SSG Attendees

**Fig. 1:**
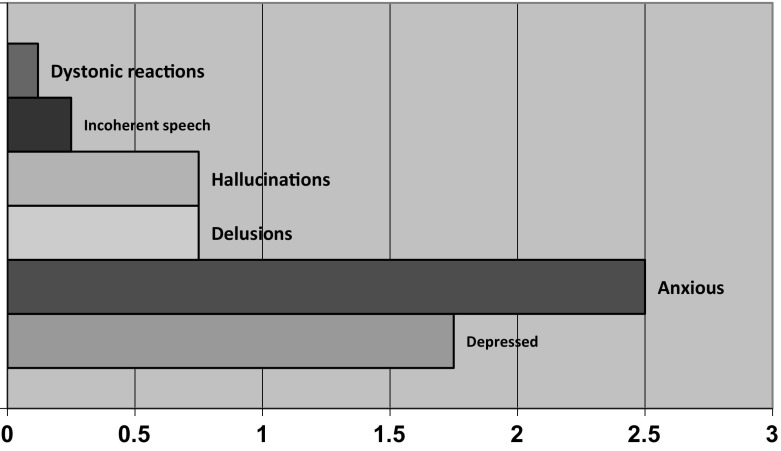
Krawiecka scores by individual factors (*mean scores high* = *poor behaviour)*

Krawieka Rating Scale (KRS) (Krawiecka et al. 1977) scores (*n* = 8) indicated that the group were generally more anxious than depressed (see Fig. 1), differing slightly from their primary diagnosis shown in Table 1 above. Two people, Peter and Kathy scored 4 (the highest score) for coherently expressed delusions and hallucinations. Interviews with these particular individuals helped validate their scores with Peter stating ‘*I get frightened of scenarios such as my connection with the mafia…I can see lights that shine and are connected with what I am thinking…electrical impulses of light going off*’, and Kathy feeling that she ‘*had special powers and abilities to be protecting other people from rapists*’. There were no signs of psychomotor retardation, or side effects of medication for the sample.

### Social Networks

**Fig. 2:**
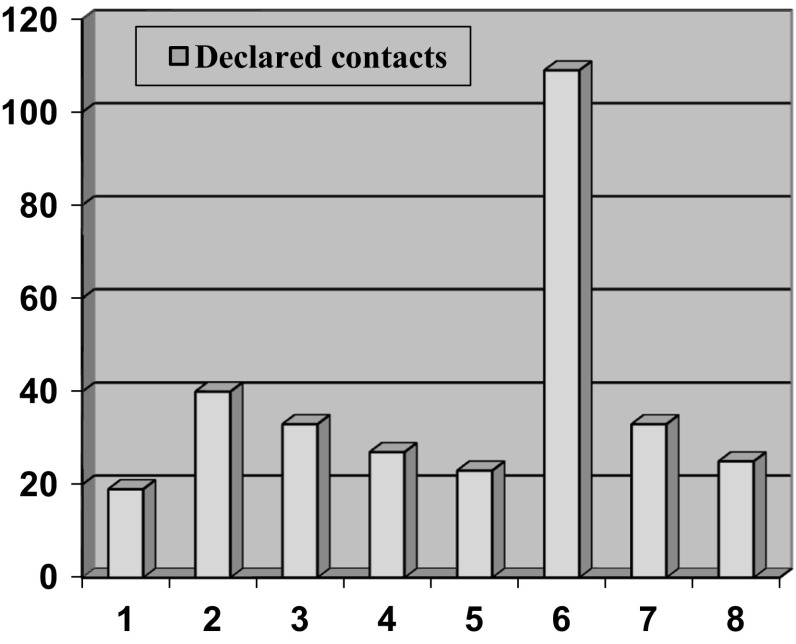
Social network size of each participant

**Fig. 3:**
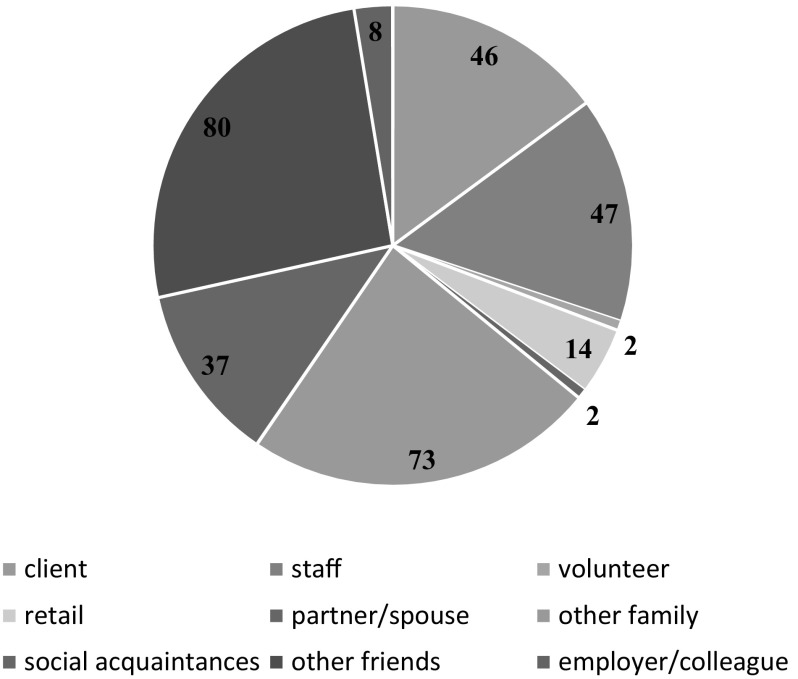
Distribution of social network membership (*n* = 309 for total group)

We had data pertaining to social networks for 8 participants (see Fig. 2). The mean network size was 39 contacts (range 10–109) decreasing to 33 (range 12–103) when staff were excluded and dropping to 28 when the score of 109 for one person was accounted for. Figure 3 shows that about a quarter (*n* = 80) of the total network members for the group were friends not associated with mental health services, whilst just under a quarter (*n* = 73) were family other than partners/spouse. However, about one-third of network members were made up of either other clients with mental health problems or staff.

### Social Support

Table 3 shows support behaviours provided by network members as well as interactions. In this study, personal support (or help with personal hygiene) was not recorded since none of the participants reported that they received this type of support. Friends appeared to be the main givers of all types of support (with each type of support provided by either one-third or just under half of all network members in each case) apart from material aid (over half this kind of support came from family members) and being critical (no one said that their friends were ever nasty to them). Family members were the next providers of support and were as critical of participants as staff (5 of those providing this behaviour in each case). There was also some evidence of individuals having experienced critical or ‘nasty’ behaviour from volunteers and retail contacts (local shopkeepers) (4 of those providing this behaviour).

**Table 3:** Support behaviours provided by the various network members

Network members	Staff *N* = 47	Vol/Retail *N* = 16	Clients *N* = 46	Friends *N* = 120	Family *N* = 75	Total^a^ *N* = 304
*Support type*						
Household (*e.g. cooking, cleaning, shopping*)	7	0	8	11	5	31
Material (*e.g. money, cigarettes, transport*)	2	0	1	13	21	37
Decisions/feedback	31	14	10	59	26	140
Confiding (*secrets/worries*)	29	4	24	80	39	176
Companionship	26	16	23	112	57	234
Invisible (*keep an eye on*)	21	4	19	74	35	153
Critical (*nasty, upsetting*)	5	4	0	0	5	14
Interactional behaviours						
Reciprocal	24	10	32	110	70	246
Weekly contact	29	14	31	69	22	165
Duration (≥ 5 years)	4	8	8	39	57	116
Closeness	5	10	7	57	43	122

^a^NB network members may have provided more than one type of support

### Interactional Behaviours

Table 3 indicates that friendships were mostly reciprocal (*n* = 110 of a possible 304 network members in total) as were contacts between family members (*n* = 70), whilst relationships with staff and other clients as well as volunteers and retail contacts were reported as less reciprocal (24, 32, 10, respectively). Participants were also more likely to see their friends than any other members (69 of 165 contacts seen weekly were friends) and participants also felt closest to their friends with just under half (*n* = 57) of all friendships regarded as ‘close’ compared to just 7 other clients. Participants had known only 4 members of staff for more than 5 years reflecting high staff turnovers rather than anything else.

## Four Vignettes of People with Mental Health Problems Attending the SSG

Through the presentation of vignettes (constructed using interviews and participant observations made over a period of 1 year), an attempt was made to provide a more holistic picture of individuals’ social lives and life histories, including their experiences leading up to joining the SSG. In complementing the quantitative accounts above, it is also hoped that these vignettes will throw some light on how individuals came to attribute meaning to the SSG in relation to their mental health. Whilst a substantial amount of material was generated for some participants, others were more private or grew tired during the course of conversations (sometimes this was due to medication). The following four vignettes were chosen to represent a range of mental health experiences as well as age, gender and social network size.

### Susan

Susan was 23 years old. She lived in a house with friends during the week and stayed with one of her parents at the weekends. She had been attending the community mental health service and the SSG for the last 6 months (for three and a half days a week) and had also recently started psychodynamic psychotherapy.

Susan first saw a psychiatrist and went on medication when she was 14 years old and had been ‘on and off ill’ since then. She reported having anxiety symptoms (worrying, feeling keyed up and anxious, and having difficulty relaxing) and depressive symptoms (dwelling on the negative, lacking in energy) and engaging in self-harming behaviours. She had been diagnosed with Borderline Personality Disorder which she thought ‘doesn’t mean a whole lot really […] It’s just a label which describes a whole load of behaviours’. But: ‘What it means to me is that some of the experiences I’ve had, I’ve not done very well in processing them. So I’ve ended up in a bit of a mess’. She reported that one possible trigger for her illness might have been an episode of sexual abuse which she had experienced as a child.

Since Susan was independent in her daily life, she thought that she might soon be discharged from the community mental health team which would mean no longer having a community psychiatric nurse or being able to attend the mental health day centre. She was worried about this as she felt that she needed that support:

“If they do discharge me, it won’t be because I’m better. It will just be because they don’t have a system in place for people like me. I drive and I don’t need someone to take me shopping. I need other things, like at the moment I haven’t had umm any type of drink, since Sunday evening [three days ago], just because I’ve got it into my head that I’m not allowed to drink umm… so I need someone on my case, saying you need to do this, you need to do that. I can go to Sainsbury’s [local shop] on my own, but I mess other things up instead.”

After school she worked as a nurse for a while, and also completed a degree at university. She was now planning to start a counselling course with a view to being a counsellor. She had decided to do the course part-time so that she could continue to attend the day centre and the SSG ‘because that’s what keeps me going’ either as a service user or, if she was discharged, maybe as a volunteer.

Susan’s reported social network consisted of 19 (12 of whom she had known for more than 5 years). They included 6 staff (including 2 community mental health nurses, her key worker, her psychiatrist and two of the SSG Chaplains), 3 family, 7 friends, 2 social acquaintances and 1 client. She was in contact with 14 of these people every week and felt that 10 of her relationships were reciprocal. She was able to confide in 14 of them who also helped her make decisions. She found three of her relationships to be critical or her but said that she counted 8 of her contacts as best friends.

Concerning the SSG, Susan said:

“It’s a good opportunity to hear how other people see their spiritual selves and what impact that has on their lives. With [my] other groups, we do creative things whereas in this group, you have more of a chance to discuss things and I like [Chaplain]. He’s a good bloke.… I like it, I think it’s good, it’s quite safe…environment. Different from….I think sometimes, professionals have a very ‘us and them’ attitude, and view people with mental health problems to be needy and difficult, and just very time consuming and a bit of a pain really. I have concentration problems, but [I] can think quite clearly when [I’m] with other people although the group can sometimes stress me out as being positive makes you think too much. Sometimes I feel like staying away from other people”.

### Kathy

Kathy was 27 years old and living on her own though she saw her parents about once a month. She said she had ‘quite a troubled childhood’ and at 18 years old, she joined the ‘Jesus Army’ in London as well as a Baptist Church. She also worked for ‘Youth for Christ’ for 2 years as a volunteer after which she spent 1 year in an administrative role for the organisation. She thought that her involvement in the church possibly contributed to her becoming ill, because:

“I really got really entangled up in so much more guilt…. I’m quite a guilty person anyway, I feel guilt about lots of things. And it [involvement in the church] really intensified that. And ummm….I felt so guilty in the end – I even stopped eating for a while because…I just felt ‘I don’t deserve to eat, I don’t deserve this, I don’t deserve to do that”.

Whilst participating in church, she began to get sick and hear voices. ‘I think that was quite difficult because I don’t think they [the church] really knew what to do with me. I mean, they tried to cast out demons and stuff’. Eventually, Kathy became very sick and lost a lot of weight to the extent that her parents persuaded her to move in with them. A few weeks later she took an overdose and was admitted to hospital and for a while she was in and out of a mental health hospital. She had been diagnosed with Borderline Personality Disorder, Major Depression and Eating Disorders, but had not received a diagnosis for her acoustic hallucinations. She said that her ‘stint in hospital was useful in a way, because at that time I needed to go to a place of safety, needed to be safe although I was very quite and enclosed in [hospital ward], my anger was inside me’. After being discharged, she lived with her parents but ‘it [her illness] got too much for them’, and she moved out about 5 years ago. She says she is ‘much happier’ living on her own. Her last stay in hospital was 3 months ago. She was currently taking an anti-depressant; in the past, she had also been on anti-psychotics and mood stabilizers. She mentioned that her current medication interrupted her concentration and made answering the interview questions ‘very difficult’.

Kathy had been attended the SSG once a week for 1 year and a half. She had self-referred herself to the SSG almost as soon as she had been admitted to the hospital on a previous occasion. Now, she was a full-time client at the day centre (attending three times a week) and continued to attend the SSG. Kathy had also been attending yoga, art, swimming and cooking groups.

Her decision to join the SSG was due to her experiences with the church in London, which ‘left [her] with loads of questions, […] a bit confused’. She regarded the group as an opportunity to ‘lay things to rest and sort things out’ concerning religious questions. For example, ‘feelings of guilt over being ill, over not being able to control it [the mental illness]’ saying:

“It’s good to be able to talk about spiritual things, cause a lot of people think you’re either trying to convert them when you start talking about spirituality, or that you’re crazy, really (Laughter). So it’s good to be able to have a group of people… and everyone believes in different things, no one is like they’re all Christians, or they’re all Buddhists, you know, there’s lots of different, you know, spirituality things… so it’s kind of, it’s more open”.

She also hoped that the group sessions would help her to deal with certain past experiences and make her feel less bitter about them. She liked talking about spirituality, in a gentle way, with a group of people in which her shared experiences would be kept private. Attending the SSG, as well as other groups, was important to her because she needed to socialise, communicate, and have something to do. She also felt respected and valued in the group. Furthermore, the SSG served an interim purpose in terms of going to church, because although she felt she needed to return to it, the SSG gave her a sense of calm and helped her ‘to think about emotions, whereas I was having quite [a] struggle to differentiate between caring and not caring. It’s very easy not to care in mental health’. She did at times find herself ‘drifting off’ especially if someone else in the group ‘didn’t stop talking’ and her memory was a ‘big problem’.

What Kathy really liked about the group was that ‘You didn’t feel that religion was actually thrown at you…it helped if you got somebody you can discuss it with afterwards. It was thought-provoking’. Finally, she thought that it should have been advertised more and that better information about it should be provided so that more people would know whether or not to join.

Kathy’s social network comprised 27 members with 9 members including her parents, whom she visited about once every month. She said that her relationship with her mother was very good, but she was less close with her father. She was also in contact with a brother, a sister and five nephews and nieces. In total, Kathy was in contact with 14 of her network members each week and felt that the same 14 relationships were reciprocal. Kathy was also friendly with 7 other clients from the day centre and named 8 members of staff including 2 Chaplains. She had 3 other friends not associated with mental health services in addition. Kathy reported that 16 members in her network provided her with companionship, 11 helped her make decisions and 4 helped with household tasks though 4 were critical of her. She described 11 of her contacts as best friends 10 of whom she could confide in.

### David

David was 61 years old. He was first admitted to hospital when he was 17 years old, but said that ‘looking back I can see even in my junior school days that it was [mental illness] on the way. I tended to do things differently to other lads. I tended to isolate myself’.

After his first hospital stay, David did not return to school, but started doing various jobs instead. He describes how his mental health problems interfered with his work life:

“I got one or two jobs from [my] early twenties through to I when I was about 26, 27…but I just went off my head”.

I: right so what kind of jobs did you do?

“Mostly clerical work erm… I worked for the gas board as it was then. And I worked for the city council for about 2 years. And then I left that because things started getting on top of me; I started worrying about it and checking everything two or three times, double-checking etc. That’s all gone now by the way, I don’t do that at all. I would say, in those days the anxiety was worse than the depression. I was quite optimistic… And I would say over the years it has gone from one to the other, one has gone and the other has taken over…. And my psychiatrist didn’t want me to [work], [he] said, “get better and get a job.”… I managed to stay out of hospital until I went into Uni [University]. But relationships tended to break down. I daresay it was my fault, because I used to get depressed and needed a lot of space. I didn’t get married. I’ve been engaged once, when I was 21, for about 6 months”.

David subsequently looked after his mother for several years although he said that*: “I didn’t have a good relationship with my parents, I tended to make decisions for myself all the time. And my [younger] brother didn’t have a great relationship and doesn’t have a great relationship with me now. We didn’t do a lot together”.*

David’s social network comprised of 15 other clients, 6 members of staff including 1 Chaplain, 6 family members, 3 social acquaintances, 1 friend and 1 service contact. In relation to social support, David reported that he was able to confide in 18 people in his soical network. 17 contacts provided him with companionship and he had reciprocal relationships with 16 individuals. However, only 3 of his contacts helped him make decisions and he saw just 6 people in his social network every week.

David attended the community mental health day centre several days every week and had been attending the SSG for about 6 weeks. He joined the group because:

“it was suggested to me, there was a vacancy and would I like to come? I said Yes I would and…erm…. because I think I would get a lot out of it and put a lot into it too. It was also a course just starting, a course just starting, so it was convenient”.

Of the group he said:

“I think it has broadened my [pause] how other people see things, with spirituality and that sort of thing, how you see the world. I don’t mean in a personal sort of way but in a general sort of way. You learn that other people see it very differently and erm.. I like to learn what other people think and believe and I like to learn about other people’s lives. I can listen to people chatting about themselves for example. (Laughter) You know, about their lives and if they’ve got problems. I am good at listening … I tend to remember what people say as well. I think er that’s important. It’s no good listening if you don’t remember somebody”.

The SSG had also provided David with a structure to his week:

“Whereas before, when you don’t have any structure to your week, Monday, Tuesday… you don’t know what day of the week [it is], they’re all the same. It doesn’t really matter whether it’s Sunday or Wednesday. Whereas when you have something on a particular day it gives a sort of structure to the week. It gives a sort of pattern. You can think well it’s a Friday I will be doing such and such…and it’s interesting to meet different people and I, I, I would say [that] I have been looking for something and never found it in my life. I say that I think there is something out there but I don’t know what it is. I have been in various churches and … What I never got on very well with was what I would call, organized religion. I did try the Quakers but the only problem I had with them was their passivity and I have a problem with that…the only church I really liked but was rather small was the humanitarian one but there aren’t many. The services are very, you know, there’s no written services. We have a Minster, Pastor whatever you want to call it in the church.

I don’t anticipate things a lot. I tend to wait and see what comes out of things, rather than anticipating things. I wouldn’t say I was a happy person…I can feel good about things. I don’t get really excited, but I can feel good about things. Yes, it does. It gives me a broader idea of how other people think… because you need to deal with it. ___ especially in the last 3 years, and you tend to become very self-preserved and you can’t avoid it, it’s simply unavoidable. It comes over you, it takes you over and the longer it goes the more it hurts. I’ve been alone now for (Pause) too many years. I was alone before I looked after my mother, and then I looked after her for years….but I was available to do it, so I did it, and she was very demanding”.

### John

John was 41 years old and had been attending the day centre three times a week. John said that he has suffered from depression for a long time:

“I used to be quite active – I used to go to work and then I had an accident at work that damaged my spine and I had to give up work. And so now I am disabled and since my depression I don’t do anything I actually enjoy apart from coming here [the day cenatre] I’ve been depressed for most of my life really because […] I was abused as a child…but the thing that really set it off was my wife leaving me for a new partner 7 years ago, and, particularly, her “rejecting” their child who was then 13 years old”.

He was now living with a new partner and her child from another relationship. In total, the size of his social network was 25 comprising of 7 clients, 7 staff (including 2 Chaplains), 8 family members, 1 social acquaintance and one friend in addition to his partner. John could only confide in 1 network member (his partner) who was also his best friend and said that 2 people provided him with companionship. 3 people helped him make decisions and 25 relationships were reciprocal. He saw 24 of his social network weekly. Talking about his brother, he said: “So I have got a brother who… When I talk to my brother I feel I can’t speak to him on any level about my depression what so ever. When I tried to on a couple of occasions… On both those occasions he said, ‘what is it, you just feel sorry for yourself’”.

As regards the SSG, John said that he had not been to church ‘for quite some years now’ but that:

“talking…and especially about the spirit, it brings awareness of those feelings and thoughts that happen and it was the thought that I would be with people that would be talking….something I had not done for so, so long. You … think about things you wouldn’t normally think about. Or maybe you would have done once but not since being depressed. You start realizing certain qualities about you”.

In terms of attending the group, he said:

“To be honest with you if I had been offered [the SSG] at an earlier period I don’t think I would have attended. My depression was so bad. I couldn’t do it. I physically couldn’t do it. I didn’t go out the house… before coming here I was sitting around at home, things going through my head all the time because I get anxious. I was so desperate for somewhere to go, for something to do, to see people. Because I knew how important it was to me personally to get out… but sometimes it’s quite difficult to get out the house to come. It can be quite an event for me. There are different people within the group as well which is quite nice. Its good because on the days when you feel a little better there is also someone who doesn’t feel so good. And you start thinking how lucky you are and you help those people because you understand how they are feeling. You tend to talk to them more and they talk to you more because they get the feeling you understand and they do too”.

John also thought that other groups (like pottery) were also valuable:

“you actually make something, you don’t feel useless, it’s an achievement. I think it starts to build your spirit to repair the damage that you are not aware has been done…. It hasn’t been there for a long time, that feeling, those thoughts”

It was important for John that there were not too many expectations; ‘I’m quite aware that I don’t say that much’.

However, John reported that he had found the group to be helpful:

“I found it quite erm… early on, actually probably from the first day I came here. People were friendly. You don’t feel like you are being judged in any kind of way at all. Erm and its quite obvious that they have a positive attitude and they help you along and change your way of thinking. I think that is really important and that’s what it does for me. As I said to you earlier, I don’t really smile and when I come here I smile, I don’t understand that but I don’t feel pressurized that anything is expected. Since my depression, now I don’t do anything I actually enjoy apart from coming here. I am hoping that it’s going to help me focus clearly and in the right way on things to do with my life. I have thought that there was any way I could be a volunteer I would like to think that I can help other people if that is possible. I don’t know if it is yet.

The vignettes above demonstrate the uniqueness of individual’s lives. They also show common experiences of the SSG. Taken together with the data from the thematic analysis of the chaplains and day centre manager interviews, three main themes were derived and are presented below using quotes from the whole sample:

### Theme 1. Religious Aspect of the Group (this theme had two sub-categories):

- ‘A church substitute’;
- ‘Gentle approach taken’

All of the mental health participants reported that they ‘*enjoyed being part of the group’* and everyone stated that their motivation to join the group was ‘*seeking*’; they were hoping to ‘*find something’* that might help their recovery, as exemplified by David and, corroborated by one of the chaplains:

“I’ve been looking for something and never found it in my life’ (David)

‘There are clearly some people who are coming because they hear about what it is and have had an interest even in spirituality or in particular have some kind of nominal spiritual interest” (Chaplain 3)

Those leading the group were mindful not to allow one particular religious belief to dominate discussions, one chaplain stating:

“The main focus is not God, because we hope to have many people who are not religious…Religion is a part of spirituality obviously but spirituality is not religion. Therefore religion, God, Bible will only come up if one of the patients wants to bring it up. But then we have to be careful…because we might have atheists there as well. So you’ve got to answer their question in a much wider context of ‘a spiritual god’. Whatever religion we may be we can accept a spiritual god”. (Chaplain 1)

- A church substitute

For those participants who found formal religious services daunting, the group appeared to act as a substitute spiritual forum or at least an interim process before returning to religious ceremonies including church services. Attending large-scale religious services made some participants anxious, Simon stating “*If I can get to a state of mind where…I would like to go to church more than coming here….it won’t be so bad”*. Qualifying this, Simon said:

“Religious people have got an air about them…a kind of calmness, collectiveness, in charge of themselves and I want that, but I’m not prepared to go to church to get it so I come here…” (Simon)

(b) Gentle approach taken

Participants also reported that the SSG was led in an informal fashion which allowed for personal issues to be raised and discussed in a confidential and safe environment. That no one religion was dominant in the group was appreciated as exemplified below:

“It’s very open, very laid back” (Peter)

“You don’t feel that religion is actually thrown at you” (Helen)

“I want religion but I want it gentle, I want to be honest when I go to something. I don’t wanna feel guilty” (Simon)

“I feel respected, valued in this group” (Helen)

### Theme 2. Settling Spiritual Confusion

Six participants reported that an important aspect of the SSG was the opportunity to discuss personal matters including settling spiritual confusions as exemplified by Kathy who recounted that previous experiences of religion led to the church trying ‘to cast out demons and then I got ill. In this group [SSG] I am hoping to sort things out, lay things to rest’.

Learning about other member’s spiritual experiences was also reported as helpful:

“It’s good to be able to talk about spiritual things, cause a lot of people think you’re either trying to convert them when you start talking about spirituality, or that you’re crazy” (Kathy)

“It’s a good opportunity to hear how other people see their spiritual selves and what impact that has on their lives” (Susan)

“I think it has broadened my horizons, how other people see things, with spirituality…how you see the world. You learn that other people see it all very differently. And I like to learn what other people think and believe” (David)

It was acknowledged by the day centre manager and chaplains that whilst there were risks associated with individuals wanting to ‘sort out’ their spiritual confusions whilst mentally ‘ill’, such risks centralised around challenges to personal beliefs, and that this was not inappropriate in relation to recovery models, two chaplains stating:

“the only risk is if some people feel that thinking about belief challenges their established beliefs but then for me some things should be challenged because if people who have come into crisis and breakdown because of…the way they think and feel, then something has to change to bring them round to that so it’s looking for health…” (Chaplain 3)

“I think they appreciate the opportunity to talk and being given permission to talk about things that are important to them and…I actually think it’s also useful for them to hear what other people have to say. I think that people often go away feeling better at the end than they did at the beginning” (Chaplain 2)

### Theme 3 Helping in the Recovery Process (with two sub-categories):

- ‘feeling better’, and
- ‘structure to the week’

- Feeling better

Whilst from an objective viewpoint it was difficult to observe full recovery of the group members (at the study end, all were still NHS patients and attending the SSG), the subjective views of participants about the SSG were that it was helping them in their rehabilitation. In particular, individuals felt that the SSG sparked emotions often flattened by medication:

“it [has] helped me to think about emotion, whereas I was having quite a struggle to differentiate between caring and not caring. It’s very easy not to care in mental health” (Helen)

“I think it starts to build your spirit to repair the damage that you are not aware has been done. You can start to feel good about something you have talked about. It hasn’t been there for a long time, that feeling, those thoughts. You talk about so many different subjects […] and you think about things you wouldn’t normally think about. Or maybe you would have done once, but not since being depressed. You start realizing certain qualities about you” (John)

Participants also said they felt better having attended the group as indicated below:

“…on the days when you feel a little better there is also someone who doesn’t feel so good. And you start thinking how lucky you are and you help those people because you understand how they are feeling. You tend to talk to them more and they talk to you more because they get the feeling you understand and they do too” (John)

“I get things out of it, in fact I have got 100% better since attending these courses” (Simon)

(b) Structure to the week

Participants also recounted that the SSG offered a structure to their otherwise rather mundane week:

“It [SSG] is quite structured, which is good. I come to this group and I know where I am and that’s good” (David)

“I have nothing else to do. I need to communicate so I come here [SSG]” (Helen)

“It gives me a structure, pattern to the week and it is interesting to meet different people” (David)

“I am…desperate to come here…it is important to get out of the house, see people, something to do” (John)

Similarly, one of the chaplains commented:

“I think probably that most of them are ….initially coming because it’s better than sitting around doing nothing, so they come out of curiosity. It will kill the time between having a cup of coffee and lunch” (Chaplain 5)

## Discussion

The results from this study, that the SSG was positive in helping individuals with mental health problems feel ‘better’, tie in with the work of Swinton and Pattison (2001) who argued that spiritual support aids people who are struggling with the confusion and disturbance of their mental health problems, and Koenig et al. (2001) who argued that spirituality is positively correlated with psychological adjustment. Whereas churches and other religious organisations can act as barriers to social integration of people with mental health problems (McNaire and Smith 1998), the SSG appeared to provide a vital context, in which individuals could ask questions, discuss and ‘sort out’ spiritual issues in a softer, more piecemeal way than offered by more formal religious organisations. The group also differed from other more practice-based rehabilitation groups (e.g. gardening, cookery, art therapy) in that it made few participatory demands on people, apart from informal discussions which they could actively or passively engage with. Acting as a kind of church ‘substitute’, the SSG provided a structure to people’s day and week, a theme also discussed by Forrester-Jones and Grant (1997:101) and Forrester-Jones and Barnes (2008:169).

The SSG also enabled individuals to widen their social networks, the average network size of the group (28) comparing favourably to other studies using the same methodology of similar groups who did not attend an SSG including Forrester-Jones and Grant (1997), reporting a mean network size of 13.6; and Forrester-Jones et al. (2012), finding an average network size of 23. Nevertheless, the SSG result remains poor relative to ordinary population studies which report an average network size of 124 network members (Hill and Dunbar 2002) and corroborates Picken’s (1999) review of the literature which indicated that people with mental illnesses tend to have smaller networks than people without psychiatric disorders. The fact that the SSG was also reported to be a socially supportive group with opportunities to share experiences and ideas about spirituality may have contributed to the overall finding that the group reported their friendships, including those they formed at the SSG to be largely reciprocal. This also corroborates Sullivan’s 1992 and 1998 studies of factors that contribute to severe mental health recovery, finding that nearly half (43%) of the 46 participants interviewed identified spirituality as a resource to coping with stress, and understanding their mental illness experience but also of gaining social support. Significant correlations were found between network size and the general behaviour variables ‘*playing a useful part in things’* and ‘*feeling less worthless’*. This sense of being a part of something was also borne out in the qualitative data. It is likely then that this study sample felt a sense of belonging by simply being members of the group, which can lead to feelings of higher self-esteem, in turn, aiding recovery of mental health problems (Forrester-Jones and Broadhurst 2007).

Greasley et al*. (* 2001) suggest that whilst spirituality is important for most people with mental health problems, this is generally not matched by staff attitudes who consider other aspects of care (such as personal care) to be more important. Greasley et al, therefore advocate that multi-disciplinary training should include the area of spirituality. This was re-iterated by one of the chaplains who stated:

“I think that doctors, nurses and psychiatrists, as part of their training should all be given spirituality training. So often when they are treating an illness, they are treating symptoms. They don’t probe into a person’s background, they don’t look at the cause of the illness any more. This is what I try to do. I try to picture the cause of an illness and use that as the basis of the help I give them” (Chaplain 4)

Such training would benefit from Koenig’s (2013) text, which offers professional-specific information for nurses, clergy, mental health professionals, social workers and occupational and physical therapists as well as Taylor’s (2007) training manual which guides practitioners to talk to patients about spirituality.

Mental health specialists including social workers seem to continue to perceive the beliefs of those they care for, as not generally part of their professional remit, a link between spirituality and mental health largely unrecognised (Shafranske and Malony 1990; Snider and McPhedran 2014). Clinton (2004) recounted how she left the social work profession because although patients were expressing spiritual needs, there were no specialist services for matters of the soul. Given this, it is unsurprising that Lindgren and Coursey (1995) and Wilding (2007) found that mental health patients tended to be cautious or felt uncomfortable about mentioning their beliefs to paid staff. This paper has demonstrated how in the absence of a clear remit to include spiritual experiences within psychiatric care (see Wilding 2007), a spirituality support group can provide a useful outlet for individuals to talk about spiritual matters and gain from them in a non-stigmatising setting (Schneider et al. 2013).

## Conclusion

This study was small-scale and exploratory. It was therefore limited in that it could not indicate changes in mental state, general health and social networks as a direct result of the SSG. A future quasi-experimental study which measured pre- and post-data following an SSG intervention would allow for more concrete findings. A longitudinal study which captured the ‘journeys’ of individual’s mental health over time whilst attending the SSG would also be useful in assessing its benefits. Nevertheless, in capturing the experiences of individuals as they attend a spirituality support group, we hope to have illuminated the enjoyment and security individuals gained in coming together within a safe place to talk about matters which were important to them. The following recommendations were made by the participants of the study:

1. Caring for the Spirit groups should be more common within psychiatric services;
2. Psychiatric staff should attend the groups, so they can understand spiritual dimensions to people’s mental illnesses;
3. Continuity of spiritual care once individuals have been discharged from treatment should be accessible.

Whether or not services take up these recommendations remains to be seen.
